# Evaluating the Potential of Casein Glycomacropeptide in Adult Irritable Bowel Syndrome Management: A Pilot Study

**DOI:** 10.3390/nu15194174

**Published:** 2023-09-27

**Authors:** Yunyao Qu, Si Hong Park, David C. Dallas

**Affiliations:** 1 Department of Food Science & Technology, Oregon State University, Corvallis, OR 97331, USA; yunyao.qu@oregonstate.edu; 2 Nutrition Program, College of Health, Oregon State University, Corvallis, OR 97331, USA

**Keywords:** irritable bowel syndrome (IBS), gut microbiota, bovine kappa-casein glycomacropeptide (GMP), low-grade inflammation, dairy-derived peptide, intestinal immune markers, gut health

## Abstract

Irritable bowel syndrome (IBS) is a common gastrointestinal disorder that affects 10–15% of the global population and presents symptoms such as abdominal discomfort, bloating and altered bowel habits. IBS is believed to be influenced by gut microbiota alterations and low-grade inflammation. Bovine kappa-casein glycomacropeptide (GMP), a bioactive dairy-derived peptide, possesses anti-adhesive, prebiotic and immunomodulatory properties that could potentially benefit IBS patients. This pilot study investigated the effects of daily supplementation with 30 g of GMP for three weeks on gut health in five people with IBS. We assessed alterations in gut microbiota composition, fecal and blood inflammatory makers, and gut-related symptoms before, during and after the GMP feeding period. The results revealed no changes in fecal microbiota, subtle effects on systemic and intestinal immune makers, and no changes in gut-related symptoms during and after the GMP supplementation. Further research is needed to assess the potential benefits of GMP in IBS patients, including the examination of dosage and form of GMP supplementation.

## 1. Introduction

Irritable bowel syndrome (IBS) is a highly prevalent functional gastrointestinal disorder that impacts an estimated 10–15% of the global population [1,2,3,4]. Characterized by recurrent abdominal pain, bloating and inconsistent bowel habits, IBS not only disrupts physiological functioning but significantly hampers the quality of life of affected individuals [2,4,5].

The challenge of IBS is compounded by its multifaceted etiology. Disruptions in gastrointestinal motility, increased gut sensitivity, variations in gut microbiota, genetic factors [6,7] and numerous psychological and environmental factors [8,9] all potentially contribute to IBS onset and progression. In terms of etiology, the relationship between IBS, the gut microbiota and inflammation has recently emerged as a particularly promising axis of research. Evidence suggests that alterations in the gut microbiota composition may play a pivotal role in the pathogenesis of IBS [10,11,12]. Moreover, immune activation and low-grade inflammation have been implicated in IBS, with patients exhibiting elevated levels of inflammatory markers, such as interleukins and fecal calprotectin [13,14,15,16,17,18,19,20]. This triad of altered microbiome, inflammation and IBS symptoms underscores the potential for novel therapeutic interventions that could target these interconnected domains.

Among the potential therapeutic agents, bovine κ-casein glycomacropeptide (GMP) is a promising candidate. GMP is a 64-amino-acid peptide released during gastric digestion of dairy products containing κ-casein [21,22,23] or isolated from sweet cheese whey [24,25]. GMP possesses distinct genetic variants and undergoes post-translational modifications such as glycosylation [23,26,27,28,29]. These variations are confirmed using advanced liquid chromatography–tandem mass spectrometry methods [23,26,27,28,29]. GMP has been found to demonstrate anti-adhesive activity [30,31,32,33,34,35,36], prebiotic activity [30,37,38,39,40,41] and immunomodulatory properties [42,43,44,45,46]. Its tolerability and safety in humans have been proven in clinical trials with no significant side effects reported [47,48,49,50].

GMP’s potential benefits for IBS patients are currently under investigation, with particular emphasis on its ability to potentially modulate the gut microbiota and reduce inflammation. Initial research, including a detailed review by Qu et al. (2023), suggests that GMP might play a significant role in IBS management by potentially influencing gut microbiome dynamics, immune responses, and gut motility and barrier functions [51]. It is hypothesized that GMP consumption might alter the gut microbiota by encouraging the growth of beneficial bacterial species, although more research is needed to confirm these findings [30,37,38,39,40,41,51]. Furthermore, GMP may hold the potential to attenuate inflammatory responses commonly observed in IBS, possibly through the modulation of cytokine concentrations and the enhancement of intestinal short-chain fatty acid production, which are known for their anti-inflammatory properties [42,43,44,45,51].

In recent years, the impact of GMP feeding on human health has been examined in various clinical settings. Hvas et al. explored the implications of GMP intake in individuals with ulcerative colitis [47]. In their study comprising 24 subjects, the researchers administered 30 g of GMP daily, alongside mesalamine, for eight weeks. They observed minimal immunomodulatory effects but a decrease in gastrointestinal symptoms. (Fecal microbiota assessments were not conducted) On the other hand, in a study focusing on healthy individuals (n = 24) by Wernlund et al., the researchers administered 25 g of GMP daily over four weeks and compared the effects to skim milk [48]. Minimal changes were found in both fecal microbiota and immunomodulatory factors, and gastrointestinal symptoms remained largely unchanged. Montanari et al. examined GMP’s role in patients with phenylketonuria [49]. Their six-month study on nine subjects (including both adult and pediatric patients) employed GMP as a protein replacement. The patients’ gastrointestinal symptoms remained stable, but fecal microbiota and immunomodulatory effects were not evaluated. Lastly, Hansen et al. explored GMP’s influence on obese postmenopausal women [50]. This 13-subject study administered 15 g of GMP combined with 10 g of whey protein twice daily for a week and then three times daily for the subsequent week, resulting in a reduction in *Streptococcus* and an overall decrease in α-diversity. However, the immunomodulatory effects remained minimal, and gastrointestinal symptom evaluations were not conducted. Collectively, these studies underscore the varying responses to GMP feeding across different human cohorts.

The current investigation aimed to examine the effect of daily GMP consumption on gut health in people with IBS, focusing on changes in gut microbiota compositions, fecal and blood protein markers of inflammation, and gut-related symptoms. By analyzing these parameters, this study aims to provide a deeper understanding of the extent to which GMP supplementation can impact IBS and potentially pave the way for dietary strategies for its effective management.

## 2. Materials and Methods

This seven-week clinical trial included a one-week baseline, a three-week GMP protein powder feeding period and a three-week washout period. This study was conducted with the approval of the Institutional Review Board (IRB Number [IRB-2021-1186]) of Oregon State University (OSU), and all participants provided written informed consent. The trial is registered with ClinicalTrials.gov under the identifier NCT05482464.

### 2.1. Study Subjects

Participants for this study were identified using a variety of recruitment strategies, including the distribution of flyers, the circulation of messages on email listservs within OSU, and the distribution of news briefings from OSU’s College of Public Health and Human Science and Department of Food Science and Technology. Potential participants underwent an eligibility assessment that involved a preliminary questionnaire and a subsequent in-person interview. Eligibility criteria included an age range of 18–30 years, a confirmed IBS diagnosis, no lactose or dairy intolerance or allergies, no known gastrointestinal diseases or disorders other than IBS, no significant gastrointestinal surgeries, no habitual use of laxatives or antacids, and no intake of antibiotics or prebiotic or probiotic supplements within one month prior to the study. Out of the 61 individuals screened, 11 met the inclusion criteria. However, only five adults, consisting of one male and four females with an average age of 21.2 years, chose to participate in the seven-week clinical trial.

### 2.2. Study Interventions

This study utilized a dietary intervention that incorporated vanilla flavoring into the BiPRO^®^ GMP 9000 protein powder, which is a product of Agropur, Inc. (Le Sueur, MN, USA). This powder is composed of 74% protein, 96% of which is GMP. Each pre-weighed and pre-packaged serving comprises 14 g of this GMP powder, which includes 10 g of protein, 1 g of total carbohydrates (with no added sugars), 0 g of fat, 0 mg of cholesterol, 210 mg of sodium, 102 mg of calcium, 0 mg of iron and 46 mg of potassium. Additionally, the formulation incorporates a blend of natural and artificial flavorings. Participants received 21 packs of this supplementation at the beginning of each of the three intervention weeks. They were advised to dissolve a serving of the GMP powder in 8 oz (237 mL) of water using a blender bottle and consume the mixture in its entirety with each of their three daily meals, totaling three servings per day.

### 2.3. Study Design

The design of this study was structured into three distinct stages: a one-week baseline phase, a three-week feeding phase and a three-week washout phase.

During the initial one-week baseline phase, participants maintained their regular dietary habits without intervention. Stool and blood samples were collected on specified days for subsequent analysis, and participants were instructed to record their gastrointestinal symptoms using the Gastrointestinal Symptom Rating Scale-IBS questionnaire [52] throughout this phase.

Following the baseline phase was a three-week feeding phase, wherein participants’ diets were supplemented with GMP. The GMP supplementation involved daily consumption of three packets of GMP, totaling 30 g per day. Adherence to these guidelines was not directly measured in the study, and consumption was based on participant’s self-initiative. Participants continued to log their gastrointestinal symptoms as they had during the baseline phase.

The final phase was a three-week washout period during which participants returned to their usual dietary practices and discontinued GMP supplementation. Gastrointestinal symptoms were consistently recorded during this phase as well.

Except for supplementing with GMP during the feeding phase, participants maintained their usual dietary practices throughout the study. The study design specifically excluded the use of prebiotic or probiotic supplements and antibiotics during the study period.

### 2.4. Fecal Samples

Fecal samples from the participants were collected for two main analyses: fecal microbiota composition and fecal immune markers (calprotectin and lactoferrin). Participants were instructed to provide fecal samples on days 4 and 7 of the baseline week; on days 3, 7, 11, 14, 17 and 21 of the feeding period; and on days 7, 14 and 21 of the washout period. For the purposes of fecal microbiota analysis, OMNIgene stool collection kits (OMNIgene, Ottawa, ON, Canada) were supplied to the participants to facilitate the sample collection process. For the fecal calprotectin ELISA and lactoferrin ELISA, participants were instructed to collect a fecal sample approximately the size of a grape in a provided collection jar. While participants followed the guidelines for sample collection and initial storage, slight variability in adherence might have occurred. After collection, all samples were stored on ice with a capped duration of 12 h before being delivered to the lab. Once received, the fecal samples destined for microbiota analysis were separated into 1 mL aliquots and then promptly stored at −80 °C prior to analysis. Similarly, the fecal samples designated for calprotectin ELISA and lactoferrin ELISA were also immediately stored at −80 °C until analysis.

### 2.5. Microbiota: Extraction of DNA and Library Preparation

The fecal samples were thawed at 4 °C, and a QIAamp Powerfecal Pro kit (Qiagen, Hilden, Germany) was used for DNA extraction according to the manufacturer’s instruction. The concentration of the extracted DNA was measured using a Qubit 4 Fluorometer (Thermo Fisher Scientific, Waltham, MA, USA) and diluted to a final concentration of 10 ng/µL. The PCR assay was run for 35 cycles using 10 ng of DNA, 10 µL of AccuPrime Master Mix and 1 µL of each of the forward and reverse primers [53]. Each cycle consisted of 20 s at 95 °C, 15 s at 55 °C and 1 min at 72 °C. After the last cycle, the temperature was held at 72 °C for 5 min before dropping to 10 °C until the samples were removed from the thermocycler. Agarose gel electrophoresis was run to confirm amplification. The SequalPrep™ normalization kit from Applied Biosystems™ (Thermo Fisher Scientific, Waltham, MA, USA) was used to normalize the samples. The normalized samples were then pooled, and the DNA library was sent to the Oregon State University Center for Quantitative Life Sciences (CQLS) for microbiome sequencing using an Illumina MiSeq (Illumina, San Diego, CA, USA).

### 2.6. Microbiota: Bioinformatics

Raw sequencing data were downloaded from Illumina BaseSpace (https://basespace.illumina.com, accessed on 22 July 2022). To identify amplicon sequence variants (ASVs), DADA2 version 1.14.1 was used following the DADA2 tutorial for version 1.16 (https://benjjneb.github.io/dada2/tutorial.html, accessed on 22 July 2022). Taxonomic assignments were made using the SILVA database version 138. A phyloseq object was created and rarefied to the minimum sequencing depth with set.seed = 3. Alpha diversity (measured using Shannon or Simpson index) and beta diversity (measured using Bray–Curtis dissimilarity) were calculated using the rarefied data. An ordination plot using Bray–Curtis dissimilarity was created using the phyloseq package in R. To assess differences in microbial composition across the treatment groups and individual subjects, a permutational multivariate analysis of variance (PERMANOVA) was conducted using the Adonis function from the vegan package in R. Prior to rarefaction or normalization, mitochondrial and chloroplast sequences were removed from the data. Using phyloseq, the resulting object was filtered to include only samples with more than 5000 reads (two samples were filtered out), and ASVs were agglomerated at the genus level to reduce the number of unannotated ASVs. Highly rare genera, defined as those observed fewer than three times in at least 20% of the samples and with a relative abundance of less than 0.001%, were removed to filter out noise from the dataset. The final dataset consisted of 88 ASVs.

To identify bacterial taxa that were significantly associated with either study period or subjects, the researchers employed linear discriminant analysis effect size (LEfSe) using the Galaxy Module [54]. LEfSe is a method that combines the Kruskal–Wallis and Wilcoxon tests to identify features that are differentially abundant across groups and calculates the linear discriminant analysis (LDA) effect size to estimate the magnitude of these differences [54]. An alpha value of 0.05 was used for both tests, and a logarithmic LDA score threshold of two was applied to filter out low-abundance taxa. The study period was used as the class in the LEfSe analysis.

### 2.7. Calprotectin ELISA and Lactoferrin ELISA

The fecal samples were processed to measure calprotectin and lactoferrin levels, as these proteins are linked to inflammation in the gut [55,56,57,58,59,60,61,62] and, hence, could be monitored for a gut-specific anti-inflammatory effect of GMP. The fecal samples were processed using the Easy Stool Extraction Device (Alpco Immunoassays, Salem, NH, USA) as per the ELISA kit’s instructions. From each fecal sample, 15 mg was collected using the device and subsequently added to a vial containing 1.5 mL of universal extraction buffer. These samples were then homogenized on a shaker for 30 min. The diluted samples (1:2500 for calprotectin and 1:1000 for lactoferrin) were assayed alongside respective standard concentrations on pre-coated wells of microtiter plates, adhering to the manufacturer’s protocol. Plate readings were performed using a SpectraMax^®^ M2 plate reader, with concentrations for both calprotectin and lactoferrin being derived from their respective standard curves.

### 2.8. Blood Samples

For the purpose of measuring inflammatory cytokine markers, venous blood samples were collected weekly by a trained medical professional using 6 mL Sodium Heparin (NH) 95 USP Units Blood Collection Tubes (BD Vacutainer). After collection, the samples were immediately centrifuged at 2500× *g* for 20 min at room temperature. This process enabled the separation of plasma from the rest of the blood components. Following centrifugation, plasma was separated into 500 μL aliquots and swiftly stored at −80 °C for future analyses. These cryopreserved plasma samples were later utilized for the measurement of inflammatory cytokine markers using a multiplex ELISA technique.

### 2.9. Multiplex ELISA

Plasma cytokines and chemokines were quantified using the Milliplex immunobead assay (Human Th17 Magnetic Bead Panel, Millipore, Billerica, MA, USA) targeting the following 25 molecules: GM-CSF, IFN-γ, IL-1β, IL-2, IL-4, IL-5, IL-6, IL-9, IL-10, IL-12 (p70), IL-13, IL-15, IL-17A, IL-17F, IL-17E/IL-25, IL-21, IL-22, IL-23, IL-27, IL-28A, IL-31, IL-33, MIP-3α/CCL20, TNF-α and TNF-β. Frozen blood plasma samples were thawed on ice, and 25 μL of undiluted plasma was combined with 25 μL of magnetic bead solution in each well of a 96-well plate. After mixing, the samples, standards and positive controls underwent an incubation period on a shaker-plate at 4 °C for 16 h. Post incubation, the plates were analyzed using the Luminex 200 (Luminex Corporation, Austin, TX, USA) with the xPONENT^®^ data acquisition software (Version 4.3.309.1). The xPONENT software converts mean fluorescent intensity values to analyte concentration based on the integrated standard curve. The detection limit ranged between 2.5 and 60 pg/mL, and a five-point standard curve was generated on the plate. All steps adhered to the protocol accompanying the kit.

### 2.10. Gastrointestinal Symptom Rating Scale for Irritable Bowel Syndrome Questionnaire

Participants were prompted to chronicle their gastrointestinal symptoms using the Gastrointestinal Symptom Rating Scale-Irritable Bowel Syndrome (GSRS-IBS) questionnaire throughout this whole study [52]. The GSRS-IBS, a tool specifically designed for assessing IBS-related symptoms, encompasses five distinct symptom clusters: pain, bloating, constipation, diarrhea and satiety. Each symptom is evaluated on a seven-point Likert scale, where 1 means “No discomfort at all” and 7 means “Very severe discomfort.” A higher cumulative score reflects greater symptom severity. To ensure consistent monitoring and to capture dynamic symptom changes, this questionnaire was emailed to each subject on a weekly basis throughout the study.

### 2.11. Statistical Analysis

Statistical analysis for this study was conducted using RStudio (version 2023.03.0+386 with R 4.2.1). Given the non-normal distribution of data and the need for robust analyses that could effectively compare different phases of the study (baseline, feeding and washout), a combination of non-parametric tests—the sign test and the Kruskal–Wallis test—were employed.

The analysis was structured to scrutinize aggregated data within distinct study periods (baseline, feeding and washout) rather than based on a week-by-week breakdown. This approach was adopted to mitigate transient fluctuations and reduce the risk of errors associated with multiple comparisons. Additionally, it accommodated for occasional missing samples and facilitated the discernment of underlying biological trends that might be obscured in a more segmented analysis.

The sign test, chosen for its effectiveness in analyzing paired samples with non-normal distribution, was utilized to identify discernible differences between the baseline and feeding periods, as well as between the baseline and washout phases. This test was used to evaluate variations in cytokine and chemokine concentrations, fecal lactoferrin and calprotectin levels, scores from the Gastrointestinal Symptom Rating Scale-IBS questionnaire, and relative abundance percentage changes in taxonomic analysis. Alternatively, the Kruskal–Wallis test, a non-parametric substitute for ANOVA, was deployed for its ability to handle samples not meeting normal distribution prerequisites. This test was used to assess changes in α-diversity metrics, including Shannon and Simpson indices, and the mean number of observed ASVs across different study phases. A predetermined significance level of *p* < 0.05 was upheld throughout the analysis.

## 3. Results

### 3.1. Participant Demographics and Compliance

Out of the 61 individuals screened, 11 met the inclusion criteria. Of those, only five adults (one male and four females) elected to participate in the seven-week clinical trial. The participants were aged 20–23 years (mean: 21.2 years; standard deviation: 1.10 years) and had a body mass index (BMI) between 19.4 and 31.2 (mean: 24.58; standard deviation 4.35). Three subjects were White, one was Asian, and one was Hispanic. All participants were diagnosed with IBS-M using the Rome IV criteria.

An unforeseen event during the study was the contraction of COVID-19 by one participant during the sixth week; consequently, that person could not provide a blood sample for that week. Despite this incident and the erratic nature of IBS, this study had no dropouts. Those who were unable to provide all samples on the assigned days due to their IBS symptoms (i.e., constipation) ensured that they provided them at the earliest opportunity.

### 3.2. Microbial Diversity

**Figure 1:**
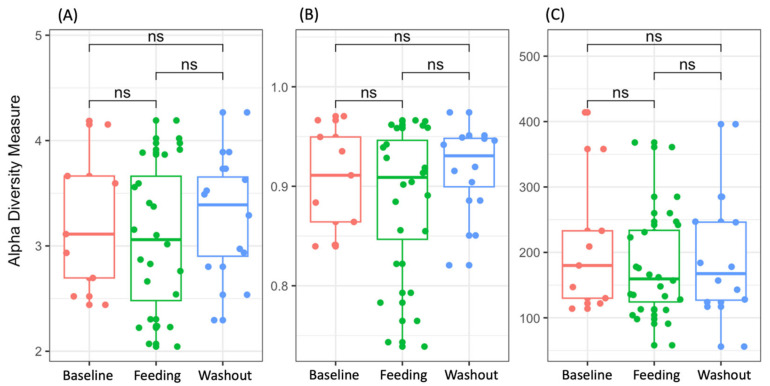
Shannon (**A**), Simpson (**B**) and observed amplicon sequencing variant (ASV) (**C**) indices for each study period. Higher alpha diversity values represent higher diversity of the microbiota. The boxes represent the interquartile range (IQR) between the first and third quartiles (25th and 75th percentiles, respectively), and the horizontal line inside the box defines the respective median. In the figure, “ns” denotes “not significant” values.

Alpha and beta diversity did not show significant differences after the GMP feeding or after the washout period. The α-diversity (Shannon and Simpson indices) at baseline was not significantly different from the value obtained during the feeding or washout period (Kruskal–Wallis test, *p*-value = 0.9403 and 0.7663, respectively; Figure 1A,B). Similarly, the bacterial richness, expressed as the mean number of observed ASVs (Figure 1C), was not significantly different across the baseline, feeding and washout periods (Kruskal–Wallis, *p*-value = 0.7388).

**Figure 2:**
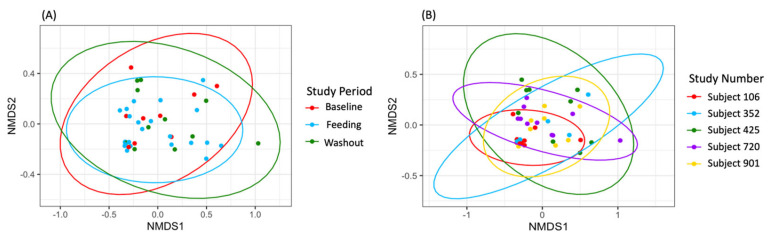
Non-metric multi-dimensional scaling (NMDS) by study period (**A**) and by subject (**B**). Sample groupings by study period using Bray–Curtis dissimilarity with confidence ellipses.

The beta diversity analysis revealed that the samples did not exhibit significant groupings by study period (R^2^ = 0.01684, *p* = 0.991; Figure 2). However, using PERMANOVA (Adonis function), the researchers found a significant clustering based on individual subjects (R^2^ = 0.15789, *p* = 0.029; Figure 2B). This observation was further supported by the non-metric multi-dimensional scaling (NMDS) plots, which indicated that the samples were primarily grouped by individual rather than by study period. These results emphasize the inherent stability and uniqueness of each individual’s gut microbiota, even in the face of dietary interventions. Furthermore, the lack of significant clustering by study period implies that the dietary intervention, GMP supplementation, did not exert a substantial impact on the overall configuration of the gut microbiota.

### 3.3. Taxonomic Analysis

**Figure 3:**
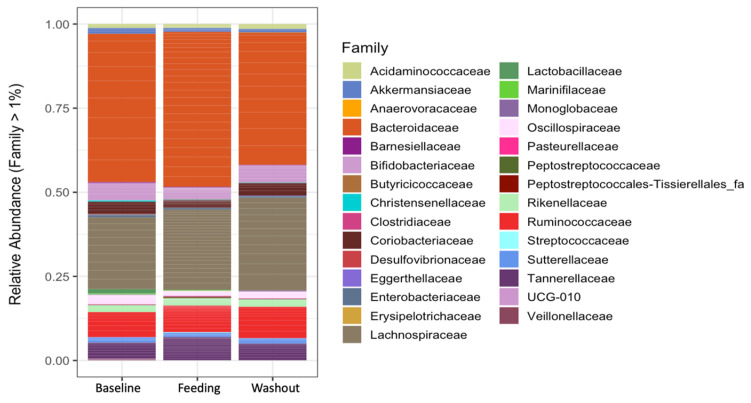
Relative abundance at the family level by study period.

The microbiota analysis did not reveal any significant difference (Sign test, *p* > 0.05) in the main (>1%) relative abundances at the phylum, family (Figure 3) or genus level when comparing the subjects over the study period (baseline, feeding and washout).

In the LEfSe analysis comparing gut microbiota across different study periods, no significant differences were observed between the baseline, feeding and washout periods.

### 3.4. Inflammatory Markers

A sign test was used to test for significant differences in the concentrations of 25 cytokines and chemokines—GM-CSF, IFN-γ, IL-1β, IL-2, IL-4, IL-5, IL-6, IL-9, IL-10, IL-12 (p70), IL-13, IL-15, IL-17A, IL-17F, IL-17E/IL-25, IL-21, IL-22, IL-23, IL-27, IL-28A, IL-31, IL-33, MIP-3α/CCL20, TNF-α and TNF-β—across the study periods. (The levels of these 25 cytokines and chemokines can be found in Table S1). Out of the 16 pro-inflammatory markers (CCL20/MIP3α, GM-CSF, IFN-γ, IL-1β, IL-2, IL-5, IL-6, IL-12p70, IL-15, IL-17A, IL-17F, IL-21, IL-23, IL-31, IL-33 and TNF-α) evaluated, only IL-15 changed significantly across the study period. Of the nine anti-inflammatory markers tested (IL-4, IL-9, IL-10, IL-13, IL-17E/IL-25, IL-22, IL-27, IL-28A and TNF-β), two markers—IL-10 and IL-4—changed significantly across the study period. These findings suggest that most of the pro- and anti-inflammatory markers remained relatively stable and unaffected by the dietary intervention.

**Figure 4:**
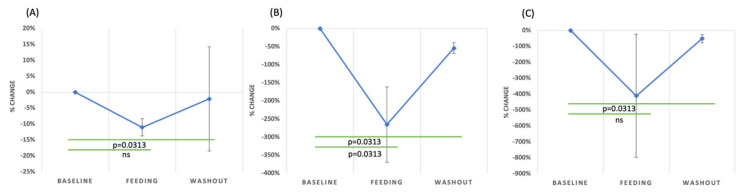
The percentage of change in the concentrations of IL-15 (**A**), IL-10 (**B**) and IL-4 (**C**) compared to baseline during the feeding and washout periods with standard error.

IL-15 is a cytokine primarily produced by enterocytes in the intestinal epithelium, and it plays a critical role in gut homeostasis, particularly by regulating intraepithelial lymphocytes. In the gut environment, IL-15 is vital for the proliferation, activation and maintenance of intraepithelial lymphocytes, which are crucial to mucosal defense [63]. IL-15 is elevated in conditions like inflammatory bowel disease [64] and celiac disease [65]. The increase in IL-15 in these conditions indicates its potential involvement in the dysregulation of intestinal homeostasis. A significant decrease in IL-15 was observed during the GMP dietary intervention (*p* = 0.0313, sign test, Figure 4A). This finding suggests that GMP has in vivo anti-inflammatory effects that could help improve intestinal health for individuals with IBS.

Numerous studies have indicated that individuals with IBS have lower levels of IL-10 than healthy individuals, suggesting a potential genetic or inflammatory component to the condition [66,67,68,69]. Because IL-10 is crucial for countering inflammation, a decrease in its levels may exacerbate inflammatory responses, which can be detrimental, especially in conditions like IBS. However, not all studies support this finding; for instance, Vara et al. reported higher IL-10 levels in IBS patients than in healthy controls [18].

Additionally, the impact of GMP on IL-10 levels has been extensively studied, but the results remain mixed. Requena et al., in their in vitro study, found an increase in IL-10 levels in rat splenocytes and Wistar rats subjected to GMP [45]. Conversely, López-Posadas et al. recorded a decrease in IL-10 in GMP-fed rats [43]. Further, Ortega-González et al. and Muñoz et al. both observed an elevation in IL-10 levels in GMP-fed mice [44,70]. Yet, E. A. Sawin et al. observed a decline in IL-10 in PKU and wild-type C57Bl/6 mice after GMP consumption [71]. IL-10 levels significantly decreased (*p* = 0.0313, sign test) during GMP feeding compared to the baseline in IBS subjects (Figure 4B).

IL-4 is a key mediator in immune regulation, specifically inhibiting the synthesis of LPS-induced pro-inflammatory cytokines and encouraging the development of Th2 lymphocytes. Vara et al.’s findings suggest that there is no significant variation in IL-4 serum levels between IBS patients and healthy individuals [18]. However, in the current study, a marked decline in IL-4 levels was observed during the GMP feeding period (*p* = 0.0313, sign test, Figure 4C). This finding is consistent with the findings by Muñoz et al., which indicated that GMP consumption in mice led to a decrease in IL-4 levels [70]. Such observations may point toward the potential role of GMP in modulating IL-4 and, subsequently, inflammatory processes in IBS.

To explore the potential anti-inflammatory effects of GMP within the intestine, fecal calprotectin, a recognized biomarker for intestinal inflammation in IBS patients [72], was measured at baseline and during the feeding and washout periods (levels of calprotectin can be found in Table S1). Calprotectin concentrations averaged 190 μg/g (SD = 382 μg/g) at baseline, increased to 285 μg/g (SD = 633 μg/g) during GMP feeding, and then decreased to 91.5 μg/g (SD = 107 μg/g) during the washout phase. One participant displayed an exceptionally high concentration during the GMP feeding phase, peaking at 2834.10 μg/g, which significantly skewed the overall data. Typical calprotectin levels in IBS patients are around 44.50 μg/g (95% CI, 32.6–141.9 μg/g) [73]. The clinical cut-off for fecal calprotectin is set at 50 μg/g [74], and values above 50 μg/g suggest the presence of intestinal inflammation. Therefore, the levels recorded during the baseline and GMP feeding periods, which were above the clinical cut-off, indicated potential ongoing inflammation. However, during the washout phase, even though the average calprotectin level decreased, it remained above the cut-off, suggesting reduced but continuous inflammation. The sign test discerned no significant difference in calprotectin concentrations across the study periods.

Additionally, fecal lactoferrin, a marker for severe gastrointestinal inflammation, was monitored throughout the study (levels of fecal lactoferrin can be found in Table S1). Elevated levels of fecal lactoferrin are associated with various gastrointestinal disorders, including inflammatory bowel disease and colorectal cancer [75]. Most participants demonstrated consistently low fecal lactoferrin concentrations throughout the study, with values predominantly at or near zero. This observation aligns with previous findings, where the median lactoferrin concentration for IBS patients was reported as 0 ± 1.4 [76]. However, one participant had notably elevated lactoferrin levels. This individual had a noticeable elevation during the baseline phase, which further increased during the GMP feeding phase, considerably surpassing the clinical threshold of 7.25 μg/g [77]. Another participant showed a transient elevation during the feeding period but returned to levels below the clinical cut-off afterward.

The analysis of fecal lactoferrin concentrations using the sign test revealed no significant variations across the study periods. These results suggest minimal intestinal inflammation in most of the cohort. The elevated lactoferrin levels observed in one participant from the outset might indicate a potential pre-existing inflammatory condition. Despite the non-significant findings from the sign test, the individual variations observed underscore the need for more detailed investigations.

In our study, GMP supplementation in IBS patients yielded mixed results regarding inflammation. While the reduction in IL-15 levels during the GMP dietary intervention suggests potential anti-inflammatory benefits, the decline in IL-10 and IL-4, which are essential anti-inflammatory cytokines, could indicate an increase in inflammation. Although fecal calprotectin levels tended to decline during washout, this trend was not statistically significant, and individual variations were notable, underscoring the differential responses among patients. Similarly, although most participants maintained low fecal lactoferrin levels throughout the study, occasional spikes were observed in certain individuals, indicating potential inflammatory challenges. In summary, while GMP shows potential anti-inflammatory properties, its effects are not consistent across all IBS subjects, indicating the need for further research to understand these differential outcomes better.

### 3.5. GSRS-IBS Questionnaire Results

From the GSRS-IBS questionnaire responses during the feeding and washout periods, it is clear that participants had varied symptom experiences. Although the sign test did not show any major symptom changes, some patterns emerged. Two out of five participants experienced increased pain during both the feeding and washout periods. Bloating became a concern for three participants during the feeding period, but during washout, two felt more bloated while two felt less so. Constipation increased for three participants during both the feeding and washout phases. For diarrhea, the feedback was mixed during feeding: two participants felt an increase and two felt a decrease, a trend that continued in the washout period. Additionally, three participants felt fuller or more satisfied during both study periods. Overall, the data point to the fact that GMP supplementation’s impact on IBS symptoms might vary from one individual to another. Some participants felt better with certain symptoms, while others did not see any improvement. This variability highlights the importance of more research to fully understand GMP’s potential benefits for IBS.

## 4. Discussion

GMP has been shown to promote the growth of beneficial bacterial strains in various animal models, including rats, mice and pigs, as well as in in vitro bacterial culture studies [30,37,38,39,40,41]. Furthermore, GMP has demonstrated anti-inflammatory effects, marked by a decrease in inflammatory cytokine levels in rats, mice and cell studies, as well as an amplification of the production of anti-inflammatory short-chain fatty acids in mouse studies [42,43,44,45].

However, previous studies in humans, including our own, show limited effects of GMP on the gut microbiome and immune markers. Our study indicates that GMP supplementation in individuals with IBS has a minimal impact on fecal microbiota composition and systemic and intestinal immune markers. Both Wernlund et al. and Montanari et al. detected no significant changes in fecal microbiota of human subjects fed GMP, which aligns with our results [48,49]. Hansen et al. reported a decrease in α-diversity among obese postmenopausal women consuming GMP [50], which differs from our results. Hansen et al. indicated that GMP feeding exerted limited immunomodulatory effects [50], which aligns with our results.

In the current study, participants exhibited diverse IBS-specific symptoms, but the changes remained statistically insignificant. Hvas et al. observed diminished symptoms in subjects with ulcerative colitis after a regimen of 30 g of GMP daily for four weeks. However, it is worth noting that a significant 37% of participants in their study demonstrated no response, hinting at GMP’s potentially varied effects [47].

The divergence in effects observed in human subjects compared to in vitro and animal models could be attributed to numerous factors including species-specific gene expression variations, especially in response to inflammatory stimuli [78], and potential differences in GMP digestion between humans and rodents [23,27]. These differences could influence the bioactivity of the resulting peptides in the gastrointestinal tract, potentially accounting for the observed disparities between rodent and human studies.

Our study’s limitations include a small participant pool and the lack of a control group, potentially affecting the direct attribution of the observed effects to GMP. The reliance on self-administration and reporting of GMP could introduce variability, and the short intervention duration might not fully capture the potential long-term effects of GMP on IBS. Future research could benefit from a larger participant base, extended intervention periods and more regulated GMP administration to render more definitive insights into GMP’s benefits for IBS patients.

Nonetheless, this study presents an initial probe into GMP’s potential benefits on IBS symptoms and fecal microbiota, setting the stage for more expansive and detailed future studies.

## 5. Conclusions

Our study on the use of GMP supplementation for IBS treatment yielded insights that challenge previous findings. There were no significant changes in fecal microbiota composition and immune markers, which was consistent with prior research, and GMP supplementation did not lead to an improvement in IBS symptoms for our subjects during the feeding and subsequent washout periods. While our data, in conjunction with previous studies, suggest that GMP’s potential therapeutic benefits for people with IBS might be limited, future studies should explore the effect of different dosages and alternative GMP formulations on fecal microbiota, inflammation and IBS symptoms.

## Data Availability

Data are available upon request.

## Supplementary Materials

The following supporting information can be downloaded at https://www.mdpi.com/article/10.3390/nu15194174/s1, Table S1: Levels of inflammatory markers in the subjects by period.
